# The Practitioner's Clinic

**Published:** 1888-01-14

**Authors:** C. R. Illingworth


### The Practitioner's Clinic.

By C. R. Illingworth, M.D., M.R.C.S.

Pyrexia and Anti-pyretics—(*continued.*)

The deficient oxygenation will have the effect also of diminishing the fibrin-forming power of the blood, and an explanation is thus furnished of the "uterine sedative" action of the drug. For it is most probable that by the liquefying action of the drug upon the blood, tension from congestion in any portion of the circulation is prevented. By *tension* is meant the state of being stretched; and the effect of such a state of the blood vessels upon the sensory nerves is pain, varying in intensity according to the amount of tension and the sensitiveness of the affected nerves. The physician is as frequently called upon to relieve tension as the surgeon. The former prescribes remedies such as ammonia, soda, the salicylates, antipyrin, &c.; the latter uses the knife, and frequently a drainage tube. According to this view of the action of antipyrin, it is a "uterine sedative" solely in virtue of securing the freedom of the circulation from local congestion of blood, and therefore from tension and consequent pain. In the same way in migraine.

Antipyrin differs from ammonia in being less stimulant—in fact, rather depressant—in its action upon the heart. The good effects recorded from the use of the drug in hæmoptysis, with doses of five grains, are rather in need of confirmatory evidence from other sources. Grave doubts have been shown to exist as to its nervine antithermic properties. It has been said to possess well-marked antiseptic power, and for this reason has been suggested as an agent in the treatment of puerperal and other septic fevers. The biniodide of mercury, however, is far more powerful as an antiseptic, and has the advantage of not reducing the fibrin elements of the blood, already in all such disorders deficient enough.

The effects of *Antipyrin* on freshly-drawn blood are the following: The hæmoglobin is dissolved out; the corpuscles consequently lose their colour, and the remaining contents shrink within the cell walls; and the tendency to form rouleaux is absent, each corpuscle, reduced much in size, standing distinct from the rest, and absolutely destitute of the slightest viscidity. The effect upon blood clot is to clarify and soften it so as to give it the appearance of gelatine. It is evident, therefore, that in common with many other drugs, but in a much more marked and wonderful degree, this drug liquefies the blood by diminishing its fibrin-forming power. It might indeed aptly be termed *antifibrin.* Its use is thus indicated in congestions of either an active or passive nature in any portion or organ of the body. Its analgesic effects are the direct result of its liquefacient action upon the congested blood, for congestion causes tension, and tension pain. Its uterine, sedative action is thus readily understood. It cures megrim by rapidly dispersing the accumulated blood in the venous radicles of certain parts of the head; and certain cases of hœmoptysis, by obviating tension in the circulation at any part of the lungs. But it promises to be of immense service in a host of other diseases dependent upon or associated with localised congestion of the circulatory system. I have found it rapidly efficacious in cases of hystero-epilepsy, after one dose of twenty grains. It will prove serviceable, if indeed it should not prove to be an actual "specific" for puerperal eclampsia, angina, asthma, and spasms of any description. In congestions of active nature, marking the onset and constituting the first stage of any purely inflammatory disorder, antipyrin is equally useful. Three days ago I was hurriedly sent for to attend a child of thirteen months, reported to be in convulsions from teething. I sent the following mixture, promising to visit the child in about an hour: Antipyrin gr. xij., syrup ½ oz. Water to one and a half ounces. One teaspoonful to be given every quarter of an hour or twenty minutes until sleep was induced. In an hour's time I called and found it dozing. The fit had lasted half an hour, but there had been none since the medicine was first given. The child had been "out of sorts" and fretful for some days, and it was cutting two teeth, the gums over which were inflamed. Temperature 102 deg., skin tending to become moist. Medicine ordered every half hour. The child was seen again in two hours, and the temperature had then fallen to 101 deg.; and again in four or five hours when the temperature had fallen to 100 deg. By the time the fifth dose was given the child got up and ran about as if nothing was the matter. At bed-time the last dose of the medicine was given, and another bottle of the same strength sent. On calling the following day the child was playing about; no medicine had been given since the last dose of the first bottle; and the temperature was 98 deg. I ordered an occasional dose at bed-time or whenever the child seemed restless or feverish, and ceased to visit it. Here we have a case of apparently commencing meningeal inflammation, rapidly cured in its initial congestive stage by the liquefying action of antipyrin upon the accumulated blood. In the acute febrile disorders of children antipyrin will prove of great service. Bearing in mind the depression and the excitement of the circulation in cases of passive and active congestion respectively, it may in some cases be desirable to combine stimulants or cardiac depressants with the drug. Aconite, for instance, has been long known to act well in cases of pneumonia of a sthenic type, and in other acute inflammatory affections likewise. Again, Ipecacuan, in acute bronchial mischief and laryngeal inflammation, by reason of its cardiac depressant properties, so rapidly and specially relieves those who suffer from these affections that it hardly has a rival. On the other hand, the value of stimulants such as brandy and ammonia is thoroughly recognised in cases of passive congestion such as megrim and other forms of headache dependent upon inherited or other cardiac and vasomoter peculiarities. Upon the general principles thus indicated, more rapid cures will probably be effected in certain cases, by stimulant or depressant treatment with antipyrin than by the use of that potent drug alone. The choice of the stimulant or depressant will, of course, depend upon the nature of the case, and the special predilection of the physician in charge.

Last, but not least, there arises the consideration of those diseases in which the use of antipyrin is *contra-indicated.* These are all the forms included in the septic group, for in them, as has already been shown, there invariably exists, sooner or later, a condition of over-fluidity of the blood. To give liquefacient remedies, such as ammonia, sodic salicylate, potassic and sodic iodide, and antipyrin, is to further the absorption of septic materials by a diffused and weakened circulation—weakened by reason of its deficient fibrin-forming power, and diffused to its own detriment, through the midst of the infective areas, on account of its greater fluidity. The efforts of the physician in treating these diseases should be directed, not to the mere treatment of symptoms, but to the eradication of the cause, and the strengthening of the blood. These can only be effected by hæmatinic and germicidal remedies, such as iron and the biniodide of mercury respectively, administered with modifications, and combined with other remedial agents according to the nature of the particular case, and the general or local sympathetic disorders of other organs or systems of the economy by which it may be accompanied.

(*To be continued.*)

